# The Use of a Diode Laser for Removal of Microorganisms from the Surfaces of Zirconia and Porcelain Applied to Superstructure Dental Implants

**DOI:** 10.3390/microorganisms9112359

**Published:** 2021-11-15

**Authors:** Anna Wawrzyk, Michał Łobacz, Agnieszka Adamczuk, Weronika Sofińska-Chmiel, Sławomir Wilczyński, Mansur Rahnama

**Affiliations:** 1 Silesian Park of Medical Technology Kardio-Med Silesia in Zabrze, M. Curie Skłodowskiej 10C Str., 41-800 Zabrze, Poland; anna.wawrzyk@gazeta.pl; 2 Chair and Department of Oral Surgery, Medical University of Lublin, Chodźki 6 Str., 20-093 Lublin, Poland; rahnama.m@interia.pl; 3 Institute of Agrophysics PAS, Doświadczalna 4 Str., 20-290 Lublin, Poland; agn.adamczuk@gmail.com; 4 Analytical Laboratory, Faculty of Chemistry, Institute of Chemical Sciences, Maria Curie Skłodowska University, Maria Curie Skłodowska Sq. 2, 20-031 Lublin, Poland; wschmiel@umcs.pl; 5 Departament of Basic Medical Science, Faculty of Farmaceutical Sciences in Sosnowiec, Medical University of Silesia, Kasztanowa Str. 3, 41-200 Sosnowiec, Poland; swilczynski@sum.edu.pl

**Keywords:** diode laser, implant-prosthetic, superstructure, microorganisms, porcelain, zirconia

## Abstract

The aim of this paper was to study the effectiveness of a diode laser (LD) for removal of microorganisms isolated from porcelain and zirconia crown surfaces used in implantoprosthetics in order to minimize infections around dental implants. In order to optimize biocidal efficacy of the process (at the same time, avoiding increasing the surface roughness during decontamination) the effects of diode laser doses were investigated. The irradiation was performed with a diode laser at the wavelength of λ = 810 nm in three variants with a different number of repetitions (1 × 15 s, 2 × 15 s, 3 × 15 s). The quantitative microbial contamination of the surface of teeth, porcelain and zirconia crowns assessment was made using the culture-dependent method. The identification of microorganisms took place using the matrix-assisted laser desorption/ionization time-of-flight mass spectrometry (MALDI-TOF MS) and next-generation sequencing (NGS) methods. The studies of the surface morphology and roughness were carried out by means of the optical profilometry, scanning electron microscopy (SEM) and optical microscopy with the C1 confocal attachment. The most important conclusion from the research is the fact that the laser operation, regardless of the exposure time, effectively eliminates the microorganisms from the surfaces used for dental implant rebuilding and does not have a destructive effect on the tested material.

## 1. Introduction

Microbiological cleanliness is an extremely important element during implant-prosthetic treatment procedures, contributing to their success. There are a number of microorganisms in the human mouth, such as bacteria [1], viruses [2], fungi [3] and protozoa [4]. These microorganisms include pathogens which cause inflammation, affecting the time of wound healing. Villar et al. [5] found that reducing the number of bacteria on the implant surface diminished implant failure. It should be also noted that as a consequence of the violation of tissue continuity during the prosthetic implant procedures, microorganisms can enter the bloodstream. As a result, various types of complications can take place, resulting in a great risk in the case of immunocompromised patients [6,7,8].

The process of microorganisms’ colonization in the oral cavity depends on many factors such as the redox potential (Eh), the presence of antimicrobial agents and pH value [9]. An important factor is also the roughness of the surface inside the mouth which has a large impact on the initial adhesion and retention of microorganisms [10,11,12,13]. Chevalier et al. [14] described the bacterial deposition mechanism. In the first stage, adhesion to hard or soft tissues occurs. Then, matrices are formed (due to the microorganisms’ co-adhesion and coaggregation processes) and then during maturation the quorum sensing system develops. In the final stage, the devastated tissues are detached. The bacteria related closely to each other and to the solid support (through the exopolymer matrix in which they are embedded) form a metabolically integrated community of microorganisms called a biofilm [15]. The creation of a quorum sensing system enables better protection of bacteria against the host’s defence mechanisms and intensive expansion on the surface of the oral tissues [16,17,18]. 

Biomaterials used for oral restoration are much more susceptible to biofilm creation than teeth containing naturally biocidal substances [19,20]. Lee and Wang [21] reported that materials with a roughness of Ra > 0.2 intensify biofilm formation. Therefore, apart from the requirements for roughness, the materials used in the implanted prosthetics must also meet the criteria regarding biocompatibility, tensile and compressive strength, hardness and stiffness.

There are a number of different types of materials used for building up dental implants. Currently, the most commonly used for this purpose are porcelain [22,23] and zirconia [24,25]. Zirconia (ZrO_2_) is a potential substructure material. It is characterized by great mechanical properties (compression resistance of about 2000 MPa). ZrO_2_ stabilized with Y_2_O_3_ (yttrium tetragonal zirconia polycrystals) has a fracture toughness twice or even larger than that of alumina ceramics [19]. Due to its excellent biocompatibility, properties and esthetics it has become a common material for dental restorations [26]. Porcelain is a translucent white ceramic fired to a glazed state. The production process mainly influences the porcelain’s roughness [19]. It can be classified as an ultra-low fusing, low fusing, medium fusing and high fusing type. 

In order to reduce the number of microorganisms, non-surgical methods such as mechanical cleansing, antiseptic therapy and antibiotic therapy are most often used in implanted prosthetics [27,28,29]. One of the non-surgical methods of decontamination is also the use of a diode laser. However, this process must be in a way that, while it maintains the desired biocidal effectiveness, does not adversely affect the structure of the irradiated material [30].

The aim of this research was to assess the effectiveness of using a diode laser in the removal process of microorganisms isolated from the surface of materials used in implant prosthetics used for the superstructure of crowns-zirconia and porcelain with simultaneous determination of the effect of decontamination. Using matrix-assisted laser desorption/ionization time-of-flight mass spectrometry (MALDI-TOF MS) and the next-generation sequencing (NGS) method, qualitative and quantitative analyses of the microorganisms on the surface of teeth, zirconia crowns and porcelain crowns were made before and after their irradiation with a diode laser. As mentioned above, the use of a laser for microorganisms’ removal can cause potential changes of the crown’s surface. In view of this, using such methods as optical profilometry, scanning electron microscopy (SEM) and optical microscopy with the C1 confocal attachment, the surface morphology and roughness of porcelain and zirconia were examined before and after the decontamination treatment in order to assess the extent of damage.

## 2. Materials and Methods

### 2.1. Patients and Materials

In order to obtain microorganisms for microbiological tests, swabs were taken from 10 patients aged 35–76 who did not smoke. The patients took care of their own oral hygiene, regularly brushing their teeth after each meal and using an irrigator to clean their interdental spaces. No changes, cavities, caries or pathologies were detected in the teeth during their regular dental check-ups. Implants with a zirconia superstructure were fixed in teeth positions of 36 and 37, and porcelain crowns were placed in teeth positions 46 and 47. Additionally, the microbiological contamination of healthy teeth in the immediate vicinity of superstructures was examined.

The material used for the microbiological studies included 10 superstructures of zirconia crown implants, 10 porcelain crowns (placed maximum 3 years before the research) and the surface of 10 natural healthy teeth. 

The superstructure of the Titanium Ti-6Al-4V ELI implants was made of zirconium oxide with the following composition: (IPS e.max Ceram > 60% SiO_2_, CaO, Al_2_O_3_, CeO_2_, Na_2_O, K_2_O, B_2_O_3_, ZnO, F, Li_2_O, ZrO_2_, SrO, TiO_2_ and pigments). 

The porcelain crown was made based on metal (Wirobond 280, Bego, Bremen, Germany), microopaquer (bredent, CeramBond, Poznań, Poland), Opaquer Ex3 (kurarey Noritake Dental, Tokyo, Japan) and Glaze Ex3 (kurarey Noritake Dental, Tokyo, Japan). 

During the tests of biocidal efficacy of the laser, and in order to assess the roughness and structural changes before and after irradiation, the porcelain and zirconia model materials were used. The materials were prepared in a professional prosthetics laboratory and they had the same composition as the materials used for the superstructure of the patient under examination. 

### 2.2. Assessment of Microbiological Contamination

The quantitative microbiological contamination was examined twice with a 6-month interval. The samples for microbiological tests were collected from the fragments with the area 0.4 cm^2^ of outer parts: teeth, zirconia crowns and porcelain crowns with swabbing brushes according to the ISO 18593:2018 standard [31].

The collected samples were shaken in 10 mL of sterile saline and, from the obtained initial suspensions, 10-fold dilution series were made. Then, 0.3 mL of serial decimal dilutions was used to inoculate the culture media (Columbia Blood Agar, Tryptic Soy Agar and Sabouraund Agar, Oxoid, Różnowo, Poland). The plates were prepared in triplicate and incubated at 37 °C for 48 h (bacteria) and 5 days at 30 °C (fungi). The colony forming units (CFU) were counted for each replicate and the results of quantitative analyses were presented as the arithmetic means of CFU detected on all plates inoculated with the same initial suspension (CFU/0.4 cm^2^ of the tested area). Then, qualitative analyses were performed using two methods. The identification of individual bacterial and fungal strains cultured on the agar plates was performed by the matrix-assisted laser desorption/ionisation time-of-flight mass spectrometry (MALDI-TOF MS) method using the Microflex LT system (Bruker Daltonics, Bremen, Germany) and the IVD HCCA matrix (Bruker, Billerica, MA, USA). The procedure for preparation of each colony of microorganisms included direct transfer of a small amount of culture to the test plate and application of the Bacterial Test Standard control and matrix. Determination of microbial species was made by comparison with the MBT Libraries. The identification of bacteria and fungi from the mixture of microorganisms isolated directly from the test surfaces took place by the next-generation sequencing (NGS) of the V3–V4 region of the 16S rRNA gene and the ITS1 region using the MiSeq instrument (Illumina, San Diego, CA, USA).

### 2.3. Laser Irradiation

The surfaces of porcelain and zirconia model materials with the microbial inocula were irradiated using an LD (Elexxion claros AG, Singen, Germany) with a fiber diameter of 600 µm at the wavelength of λ = 810 ± 10 nm. L-mode 25 W/PM:15,000 Hz/10 µs/3.84 W pulses were delivered from the laser in the surgical peri-implantitis configuration. Radiation was delivered in three variants differing in their number of repetitions, namely L1: 1 × 15 s, L2: 2 × 15 s, L3: 3 × 15 s, with 1 min intervals between the consecutive exposures.

### 2.4. Assessment of Decontamination Effectiveness 

The biocidal efficacy of laser irradiation was determined based on the number of microorganisms isolated from the test surfaces and identified down to the species level. The tests were carried out on the species of microorganisms that were most abundant on the tested surfaces. One of the isolated species of the genus *Rothia* was selected. In addition, a *Staphylococcus aureus* strain from the established American Type Culture Collection ATCC culture was used. The microorganisms were suspended in the sterile physiological saline to obtain inocula at the concentrations of 10^6^ CFU/mL. Aliquots of 50 µL were applied to various materials (porcelain, zirconia and tooth) in a manner allowing them to dry out and to prevent spillage.

Laser radiation was applied in three variants (as described above) and its biocidal efficacy was assessed. The percentage reductions in the microbial counts (R%) were calculated according to the following equation:

R% = ((N_0_ − N)/N_0_) × 100% (1)

where N_0_—the microbial counts before exposure and N—the microbial counts after exposure (determined in the agar and sheep blood cultures, where the culture plates were incubated at 37 °C for 48 h).

### 2.5. Statistical Analysis

In order to calculate the standard deviations and arithmetic means as well as to evaluate statistical significance of differences, the experimental data for the numbers of microorganisms were analyzed statistically using ANOVA. The least significant difference (LSD) test was carried out at the significance level of *p* < 0.05 by means of the Statistica 6.0 software (Statsoft, Tulsa, OK, USA). 

The roughness measurement uncertainty, Ra, was estimated taking into account repeatability, recovery, de-calibration of the apparatus and pattern uncertainty. The uncertainty was estimated for the two extreme points of the Ra measurement range (upper and lower limits) with the assumption of a trapezoidal distribution.

### 2.6. Optical Microscopy

The surface imaging of the tested materials was performed using the Eclipse MA 200 (Nikon, Tokio, Japan) optical microscope with the D-Eclipse C1 confocal attachment. Micrographs of the examined objects were made in reflected, polarized light. Fluorescence images of the same objects were also made using a confocal attachment with a violet laser with a wavelength of λ = 405 nm.

### 2.7. Scanning Electron Microscopy (SEM) 

Scanning electron microscopy (SEM) was used to examine the surface morphology of control samples (untreated) and those irradiated with the diode laser in three variants. Prior to the SEM imaging, the samples were deposited onto aluminium specimen mounts with self-adhesive carbon conducting discs. To increase the topographic contrast and to eliminate electrostatic loading of samples during scanning, they were coated with a thin Au conductive layer using the EM SCD005 sputter (Leica, Wetzlar, Germany). The microscopic images were made by means of the FEI Quanta 3D FEG scanning microscope (Thermo Scientific, Waltham, MA, USA). The imaging of the surface microstructure topography was obtained at magnifications of 5000×. Due to the nature of the tested materials, the SEM images were made in high vacuum and at low accelerating voltage. 

### 2.8. Optical Profilometry

Roughness measurements of the tested materials were made using the Contour GT-K1 optical profilometer (Veeco, Plainview, NY, USA). The tests were carried out using the VSI (Vertical Scanning Interferometry) technique, where the maximum scanning height was 10 mm. This method uses a wide spectral range of light. A measurable difference in the height between the adjacent pixels is ¼ of the light length. The roughness parameters were determined for three areas of the test samples for the following scan sizes: 117.2 × 156.3 µm, 46.9 × 62.5 µm and 1261 × 946 µm. From the obtained results, the mean value for the Ra parameter was calculated. In order to determine the roughness parameters correctly, the sample inclination was corrected and the waviness of the surface was taken into account.

## 3. Results

### 3.1. Assessment of Microbial Contamination of Zirconia Crown, Porcelain Crown, and Tooth Surfaces

The average microbial contamination of the tested zirconia crowns, porcelain crowns and teeth surfaces ranged from 10^5^ to 10^7^ CFU/0.4 cm^2^ (Table 1). The results showed that the greatest microbial counts were found on zirconia surfaces. During the initial test it was found that the porcelain surfaces were characterized by the smallest colonization rates. The analysis of microbial contamination after 6 months indicated that the microbial counts were higher compared to the initial test, although the order was somewhat different: again, zirconia was characterized by the highest microbial counts while the lowest counts were observed on the tooth surface (Table 1). The largest difference in the bacterial counts between the tests carried out with a 6-month interval was observed for the porcelain crown surface. The largest number of microbial colonies was determined on the zirconia crown surface. 

The most common microorganisms (identified by MALDI-TOF MS in the agar and sheep blood cultures included) were *Rothia dentocariosa*, *Rothia aeria*, *Neisseria subflava* and *Streptococcus pneumoniae*. The other microbial species identified in this test were less abundant. The largest numbers of CFUs of the four microbial species as identified by the MALDI-TOF MS technique were found on the zirconia crown surfaces. The colonies cultured from the tooth surfaces were less abundant than those cultured from the porcelain crown surfaces (Table 2).

As can be seen from Table 2, the most common microorganisms as identified by MALDI TOF MS, *N. subflava*, were not detected on the tooth surfaces. *S. pneumoniae* was the most prevalent species and it was characterized by the highest counts being detected on the zirconia crown surfaces. The smallest CFU values were observed for *R. aeria* on the tooth surfaces. 

For greater identification and assessment of microbiota diversity, the metagenomic studies were carried out on the collected samples. The NGS analysis revealed that 99.99% of the obtained sequences 16S rRNA identified on the zirconia crown surfaces were classified within the kingdom of bacteria while 0.99% of the sequences ITS1 matched those typical of the fungi kingdom (108,777 and 125,257 readouts, respectively). On the porcelain crown 100% of the sequences 16S rRNA were classified within the kingdom of bacteria while 3.2% of the sequences ITS1 matched those typical of the fungi kingdom (119,038 and 76,720 readouts, respectively). For comparison, 99.99% of the sequences 16S rRNA identified on natural tooth surfaces were classified within the bacteria kingdom and 12.79% of the sequences ITS1 matched those typical of the fungi kingdom (103,247 and 79,086 readouts, respectively). The analytical pathway facilitated matching of the dominant groups of sequences as identified on all types of surfaces down to the family level or in some cases to the species level. The tested surfaces of crowns and teeth were colonized by 12 strains of bacteria identified to the species level and 22 strains of bacteria identified to the genus level. Ten fungal species were identified on all studied surfaces. Among fungi 10 species were identified in all plots. In addition, Enterococcaceae, *Streptococcus* sp. and *Rothia* sp. were most prevalent on the tested surfaces (Table 3). Operational taxonomic units (OTUs) with the relative abundance of <2.00% included 27 bacterial taxa and 6 fungal taxa for zirconia crowns, 16 and 7 taxa for the porcelain crowns and 13 and 6 taxa for the tooth surfaces. 

Accurate identification using the NGS method revealed the presence of Actinomyces, *C. durum*, *Granulicatella*, *H. parainfluenzae*, *N. subflava*, *R. aeria*, *R. dentocariosa*, *R. mucilaginosa*, *Streptococcus* spp., and *V. dispar* on all surfaces. 

The presence of bacteria strains from the genus *Bacillus* and the family Planoccocaceae were found only on the surfaces of natural teeth while those from the genus *Kocuria* were found only on the surfaces of porcelain crowns. The bacteria from the family Enterococcaceae were present only on the surfaces of the zirconia and porcelain crowns. The bacterium *S. aureus* was detected on the surface of teeth and porcelain crowns. The largest variety of microbial species (not identified on other surfaces) was observed on the zirconia crowns. These included microorganisms from the genera: *Abiotrophia*, *Atopobium*, *Campylobacter*, *Capnocytophaga*, *Leptotrichia*, *Porphyromona*, *Rickettsiales*, *Streptophyta* and species *P. melaninogenica*, *P. pallens* and *S. anginosus.* There were no *Enterobacteriaceae* bacteria on the surface of the porcelain and the tooth, the presence of which was detected on the zircons. All areas were inhabited by *Corynebacterium durum.*

On the surfaces of all tested materials, the OTUs detected with the relative abundance of >2.00% included the bacteria of genus *Streptococcus* and species *R. mucilaginosa*, and the fungal species of *M. restricta*, *M. pinicola*, *N. ramulariae* and *P. thomii.*

Additionally, OTUs detected with a relative abundance of >2.00% on the surfaces of crowns and teeth included the bacteria of genus *Bacillus* and family Enterococcaceae, species *S. aureus* as well as the fungi of the genus *Cladosporium* and those of species *A. pullulans*, *P. sporulosa* and *T. gibbosa*.

As follows from Table 3, the identified microorganisms included pathogens or potential pathogens of the mouth such as *Actinomyces, Fusobacterium, P. melaninogenica, P. nigrescens, P. palens* and *S. anginosus*. As for fungi, the pathogenic species *M. restricta* was detected in the study plots.

### 3.2. Evaluation of the Biocidal Effect of Laser Radiation 

All exposure repetition variants led to a reduction in the counts of all tested microorganisms as compared to the unexposed samples of zirconia crowns, porcelain crowns and natural teeth surfaces (Table 4). Unexposed, inoculated samples of all materials had bacterial counts at the level of 10^5^–10^9^ CFU/0.4 cm^2^. 

The counts of *N. subflava* and *R. dentocariosa* as inoculated on the zirconia, porcelain and teeth surfaces decreased in proportion to the number of repeated exposures. The same dependence was observed for *S. pneumoniae* on porcelain and *S. aureus* ATCC on zirconia. The greatest reduction in the number of microorganisms was observed on all surfaces after exposure with the L3 dose, i.e., the triplicate exposure to 15 s radiation pulses at 1 min intervals. Disinfection efficiency greater than 90.0% was obtained for 7 out of 12 samples irradiated with the L3 dose.

In the case of *S. pneumoniae*, the exposure was most efficient in reducing the microbial counts on the zirconia surfaces whereas for the remaining tested microorganisms better reduction was found on the porcelain surfaces. As regards the porcelain crown surfaces, reduction of more than 90% was found for *N. subflava* and *S. aureus*. Concerning the zirconia, this reduction level was observed for *S. pneumoniae* and *S. aureus* ATCC. *S. aureus* ATCC proved to be the most susceptible to triplicate irradiation both of zirconia and porcelain surfaces. Of all trials of triplicate exposure, the smaller reduction was for *R. dentocariosa* on the zirconia crowns. The smallest reduction efficacy on porcelain was observed for S. pneumoniae. Of all tested materials, the tooth surface was found to have the highest percentage reduction of bacterial counts for all microorganisms except *N. subflava*. The complete elimination of microorganisms was only achieved after irradiation of the tooth surface inoculated with *Staphylococcus aureus* ATCC. 

Since the best reduction efficacies were observed for the triplicate irradiation, translating to the longest overall exposure, porcelain and zirconia crowns irradiated according to this regimen were selected for further imaging studies and surface coarseness tests.

### 3.3. Surface Morphology Analysis

#### 3.3.1. Optical Microscopy

**Figure 1:**
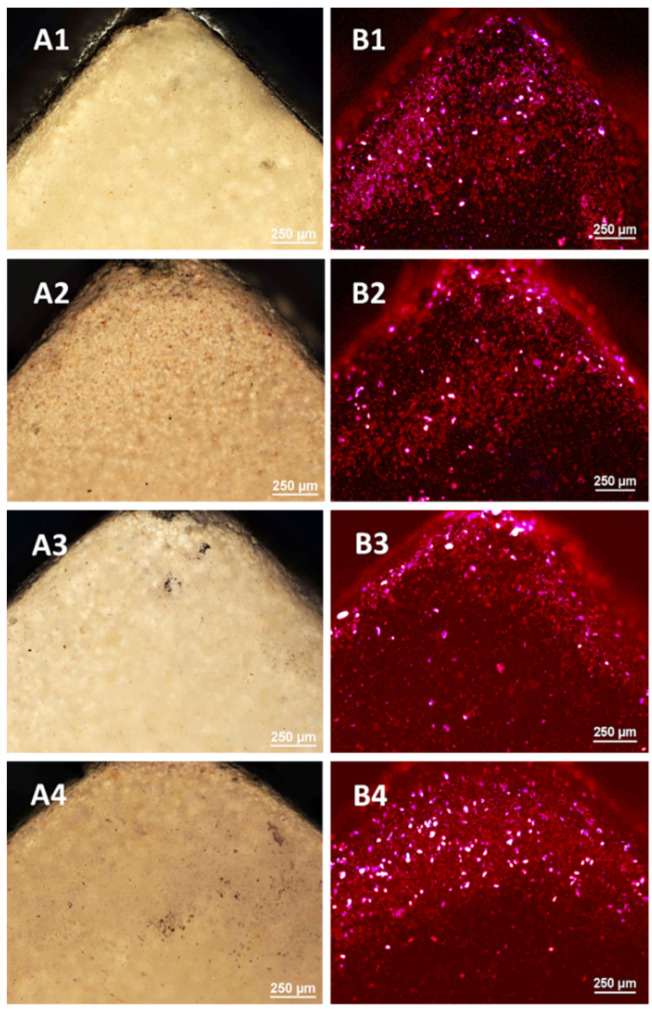
The micrographs of the surface of the porcelain samples made with the use of an optical microscope: (**A1**) the control sample; (**A2**) after irradiation with L1; (**A3**) after irradiation with L2; (**A4**) after irradiation with L3. Microphotographs taken from the surface of a porcelain sample using a confocal microscope with a laser wavelength of λ = 408 nm: (**B1**) the control sample; (**B2**) after irradiation with L1; (**B3**) after irradiation with L2; (**B4**) after irradiation with L3.

**Figure 2:**
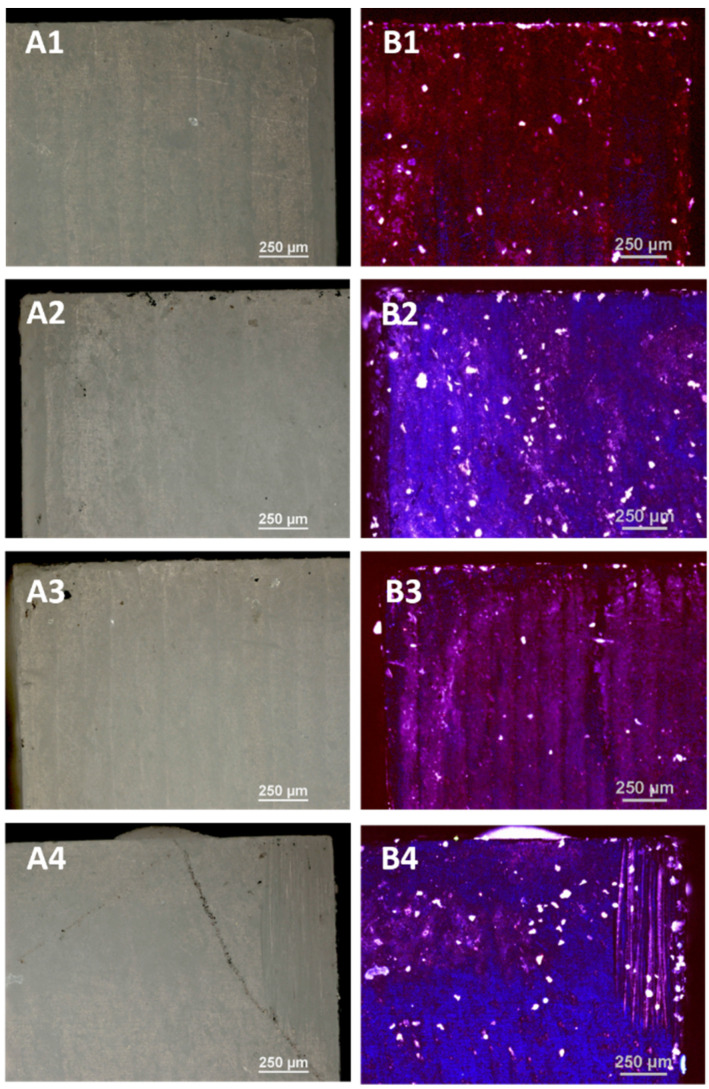
The micrographs of the surface of the zirconia samples made with the use of an optical microscope: (**A1**) untreated control sample; (**A2**) after irradiation with L1; (**A3**) after irradiation with L2; (**A4**) after irradiation with L3. The micrographs made from the surface of the zirconia sample with the use of a confocal microscope with a laser wavelength of λ = 408 nm: (**B1**) the control sample; (**B2**) after irradiation with L1; (**B3**) after irradiation with L2; (**B4**) after irradiation with L3.

The control porcelain sample—(not subjected to any processing) and the samples subjected to LD irradiation in three variants (L1: 1 × 15 s, L2: 2 × 15 s and L3: 3 × 15 s, with 1 min intervals between consecutive exposures) were examined using optical microscopy and a confocal attachment. The test results are presented in Figure 1 and Figure 2.

The tests carried out using optical microscopy did not show any destructive effects on the porcelain surfaces after the application of all variants of irradiation.

The images of the violet light of the laser show the overall fluorescence of the surface which is characterized by a red colour, changing to a brighter one with the dose of radiation. The sample B4 shines the brightest after applying irradiation 3 × 15 s. On the surface of the tested samples there are objects (grains) glowing with different colours due to different levels of laser radiation absorption by different materials. These tests showed the greatest homogeneity of the tested samples.

The tests carried out using optical microscopy did not show any destructive effects on the surface of the zirconia after applying all disinfection variants as in the case of porcelain. The 408 nm violet laser light images show the overall blue fluorescence of the surface which changes with the radiation dose to a brighter pure colour. The images of the laser light show the highest fluorescence for the samples D2 and D4. The bright white dots are the result of the resin filler grains fluorescing.

#### 3.3.2. Scanning Electron Microscopy

The SEM pictures were made in order to evaluate the nature of the changes in the chemical structure as well as to examine the topography and morphology of the control samples (untreated) and the samples irradiated with diode laser in three variants 

**Figure 3:**
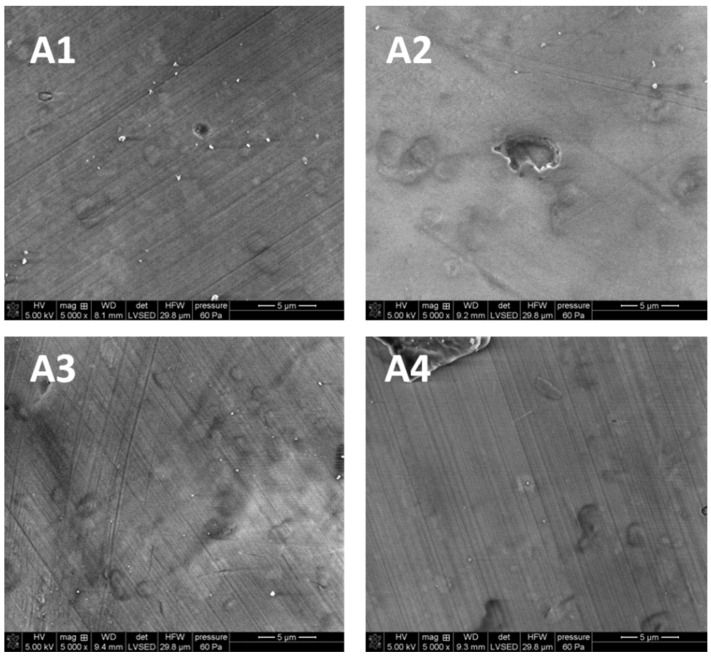
The SEM images of porcelain (5000×): (**A1**) untreated control sample; (**A2**) after irradiation with L1; (**A3**) after irradiation with L2; (**A4**) after irradiation with L3.

As shown in Figure 3 the surface of porcelain is smooth and slightly pitted. It was found that the greater the laser dose, the fewer visible scratches can be found on the surface in the samples treated by LD as compared to the control sample. 

**Figure 4:**
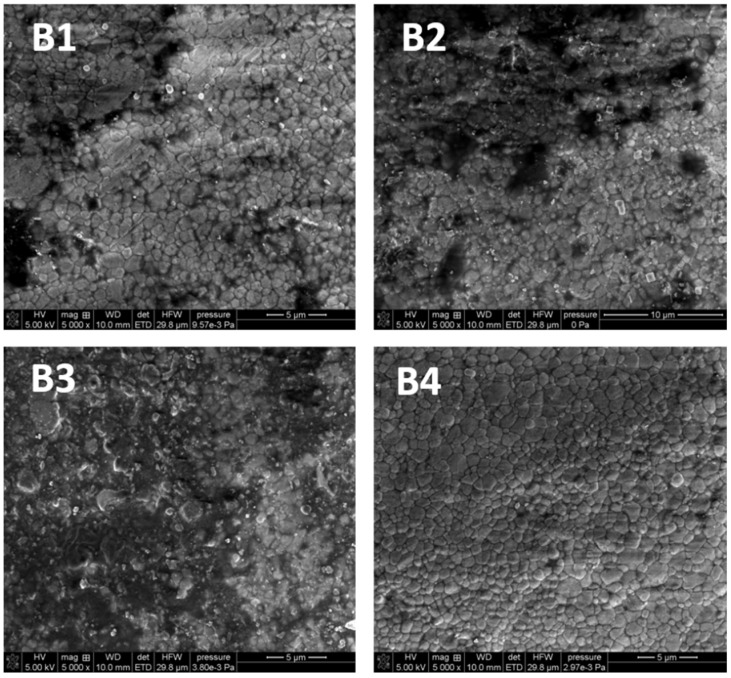
The SEM images of zirconia (5000×): (**B1**) untreated control sample; (**B2**) after irradiation with L1; (**B3**) after irradiation with L2; (**B4**) after irradiation with L3.

As follows from Figure 4, there are no differences in the surface morphology of the tested samples. The comparison shows that the larger radiation dose, the fewer black spots on the surface of the material can be seen. The images show the cleaning effect of the laser, especially in the case of variant 3. To sum up, the SEM images of porcelain and zirconia proved that their structures differ significantly. The tests did not reveal any destructive effects of laser irradiation on the surface of the tested material, regardless of dose. It can be stated that the surface of the tested materials remains unchanged after the laser application.

#### 3.3.3. Optical Profilometry

**Figure 5:**
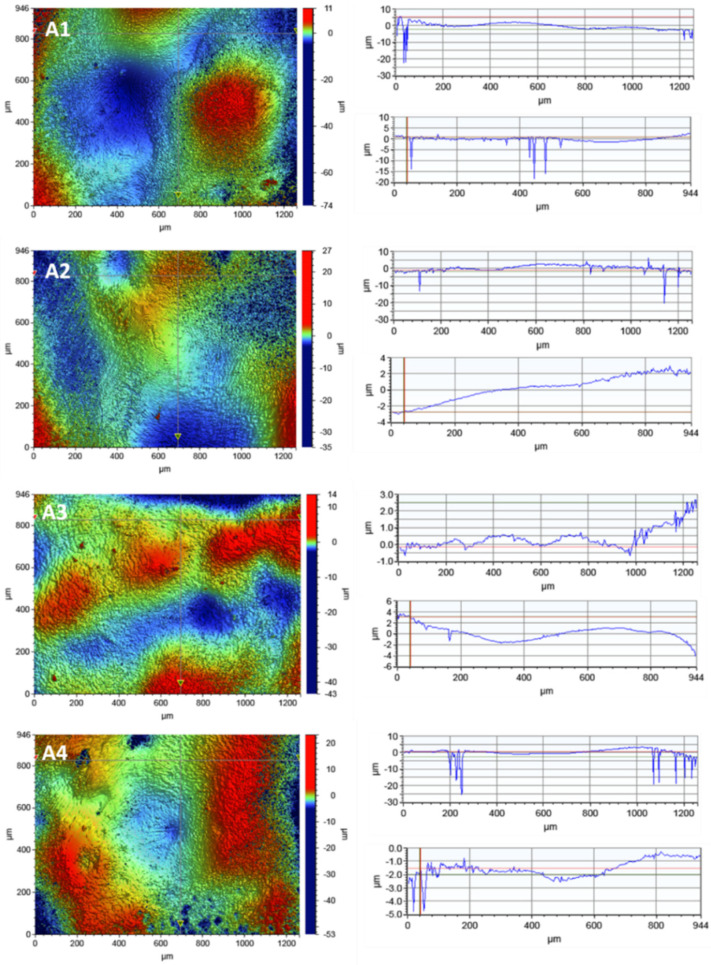
The surface microgeometry maps made of porcelain: (**A1**) untreated control sample; (**A2**) after irradiation with L1; (**A3**) after irradiation with L2; (**A4**) after irradiation with L3 and their height profiles made in the x-axis (upper profile) and y-axis (lower profile).

In order to observe the porcelain surface before and after the disinfection process, tests were carried out using optical profilometry. Figure 5 presents the 2D maps of the surface topography made for the areas of 1261 × 946 µm. Table 5 presents the selected roughness parameters for all subjects of the scan sizes: 117.2 × 156.3 µm, 46.9 × 62.5 µm and 1261 × 946 µm.

The results did not show any destructive effects of the irradiation process on the porcelain surface.

The surface roughness did not increase as a result of irradiation with the laser diode. However, a decrease in the roughness parameter Ra was observed after the irradiation process in all variants.

No linear dependence of the decrease in the roughness parameters with the disinfection time was observed. The linear profiles in the x-axis and y-axis show local depressions in the material with a depth of up to 25 µm.

**Figure 6:**
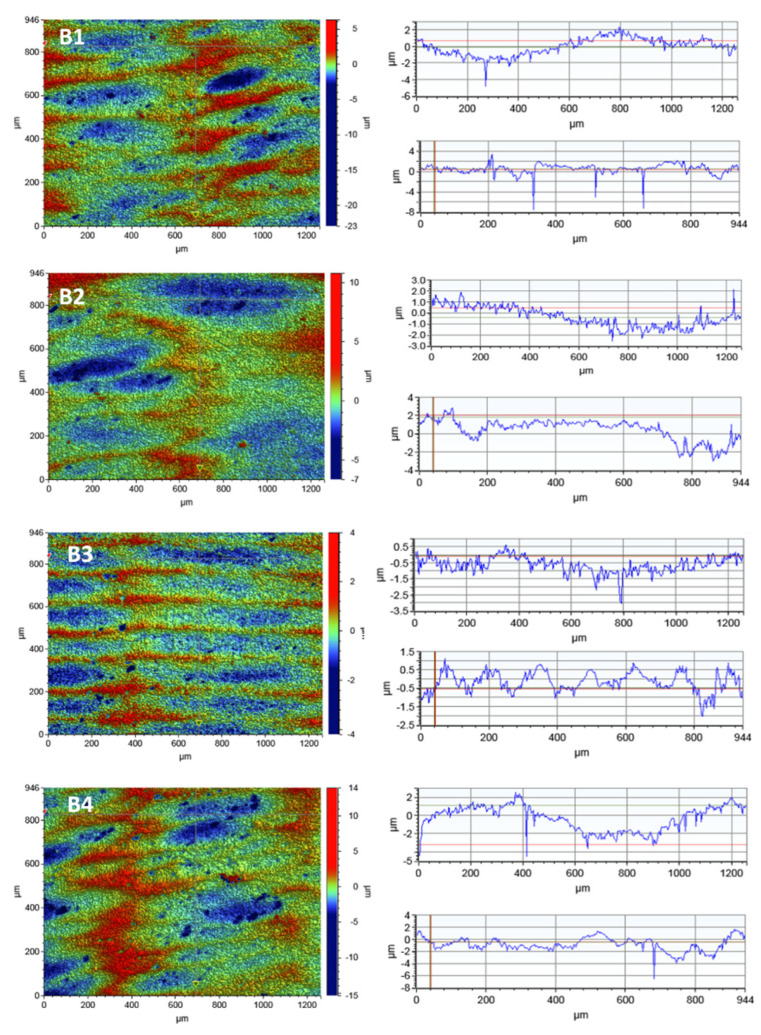
The surface microgeometry maps made for zirconia: (**B1**) untreated control sample; (**B2**) after irradiation with L1; (**B3**) after irradiation with L2; (**B4**) after irradiation with L3 and their height profiles made in the x-axis (upper profile) and y-axis (lower profile).

Analogous studies of the surface topography were carried out for zirconia. In order to observe the zirconia’s surface, 2D maps of the surface topography were made before and after the laser irradiation. The test results for the scan size of 1261 × 946 µm are shown in Figure 6. Table 6 presents the selected roughness parameters for all tested scan sizes: 117.2 × 156.3 µm, 46.9 × 62.5 µm and 1261 × 946 µm.

The tests did not show any significant differences in the roughness parameters before and after the irradiation process. No relationship was observed between the time of irradiation and the changes in the roughness parameters of the tested material. As follows from Table 6, the mean roughness value, Ra, for a given scan size changes rather randomly which may be a result of a non-uniform surface area of the tested material.

Linear profiles made in the x-axis and y-axis showed a large number of hollows on the entire surface of the tested material which can be also seen in the SEM photos. However, these depressions are shallow and reach a depth of 8 µm, contrary to porcelain which has a smoother surface. The large depressions of up to 25 µm in porcelain increase the roughness parameters.

## 4. Discussion

Zirconia and porcelain are the materials mainly used for the superstructures of dental implants. During their use, dental plaque and biofilms form on the surfaces of artificial crowns. The biofilm includes, among others, pathogens that are harmful to human health. The less attention to oral hygiene, the more microorganisms can settle on the surface of the crowns. The higher the surface roughness, the better the conditions for their multiplication. The result of residual microorganisms can be peri-implantation inflammations, and the presence of pathogens extends the wound healing time. The tissues, which are in a direct contact with the crowns, are particularly at risk. This research showed that the zirconia surface is more heterogeneous than the porcelain surface which can favour the larger adhesion of microorganisms on its surface.

Most of the organisms in this study identified by the Human Oral Microbiome database [32] are of normal oral flora. The exceptions are *S. pneumoniae* and *S. aureus*, which are the bacterial flora of the nose, and *H. parainfluense*, which is part of the natural flora of both the nose and mouth [32]. 

The largest number of microorganism types, including the potentially pathogenic ones, was detected on the surface of zirconium crowns which indicates the predisposition of this material to the deposition of the most diverse bacterial flora. Porcelain has a much smaller species diversity of microorganisms, similar to the tooth microbiome.

On the surface of zircon and porcelain crowns, bacteria of the genera Rothia, Streptococcus, Staphylococcus, Micrococcus and Neisseria and fungi were identified, which are microorganisms that can induce diseases in humans. However, it should be remembered that diseases occur only when the balance between the host and the microbiota is disturbed. It is especially dangerous for people with reduced immunity or when the microorganism is highly pathogenic.

Oral microorganisms act metabolically jointly as a community to form an organized structure in the form of a biofilm. Among the microorganisms isolated from the tested surfaces there are bacteria of the genus Streptococcus and Actinomyces which, according to the co-adhesion and coaggregation model, are among the first to initiate biofilm formation on oral surfaces. The biofilm precursors attach to each of the examined surfaces and can be a reservoir of pathogens. Then, the attached microorganisms serve as a binding substrate for various other colonizers, including Fusobacterium sp.

Moreover, Corynebacterium durum, detected on all tested surfaces, creates a microenvironment conducive to the growth of cocci, which are particularly harmful to human health, i.e., S. aureus and S. pneumoniae. The bacterium associated with the initiation and formation of biofilms is Capnocytophaga, which in these studies was identified only on zirconium. The anaerobic Fusobacterium bacteria found on porcelain and zircons act as a bridge between early colonizers and late colonization pathogens by virtue of their ability to physically bind to these microorganisms.

*Streptococcus*, which is also abundant on the tested surfaces, grows in an oxygen-rich environment on the surface of biofilms and creates a microenvironment rich in CO_2_ and lactate with a smaller oxygen content, creating an environment conducive to the growth of other bacteria preferring the inner layers of biofilms [33].

Analysis of the types of microorganisms shows that the similarities between the surfaces of the crowns used for the rebuilding of dental implants are greater than the differences.

According to Spaulding’s classification [34], the implant is a critical element as it enters the sterile tissue and vascular system. In order to maintain microbiological safety, it should be sterilized before implantation. This is the main principle that is widely used for sterile implants in oral surgery. On the other hand, crowns are the elements that come into contact with mucous membranes, so they can be classified as semi-critical elements that should be free from microorganisms but a small number of microorganisms are allowed on them. Such surfaces should be cleaned, which can be done, among other methods, with laser irradiation [34].

Most of the pathogenic microorganisms identified in these studies can turn out to be very harmful, especially for immunocompromised patients. When tissue continuity is broken, abscess formation and tissue infections can occur as in the presence of *Actinomyces, Fusobacterium, Rickettsiales* and *Streptococcus anginosus*, which were detected on the surface of zirconia and porcelain crowns.

The laser irradiation dose selected for this study was based on a previously performed study in which 2 × 5 s laser irradiation in the manufacturer’s Periimplantitis surgical program was shown to yield a reduction of *R. aeria* on the healing abutment of 99.70–100.00% (Wawrzyk et.al., 2021). In this study, the same dose variant was tested but in three different replicates, keeping in mind the potential biocidal efficacy of the laser and the minimization of surface damage that can occur if too high a temperature is used [35]. 

The irradiation of zirconia and porcelain surfaces inoculated with the most common microorganisms in the natural environment of the oral cavity in the variant of the L3 laser, repeated two and three times in a 15 s time period, gave satisfactory results. Depending on the tested microorganism, reduction on the zirconia surface was obtained at the level of R = 99.08–99.87% for *S. pneumoniae, N. subflava* and *S. aureus*, and on the porcelain surface at the level of R = 84.57–99.94% for the same microorganisms. Dai [36] and Gutknecht [37] achieved similar reductions in the number of microorganisms using a diode laser for decontamination of *E. faecalis* and *E.coli* in root canals. Meire et al. [38] achieved reduction of *E. faecalis* of 99.8%, also in the root canal.

The main objective of this paper was to examine effectiveness of using a diode laser in the process of microorganism removal from zirconia and porcelain crowns. The consequences of this process were also considered. The evaluation of damages on the surface of superstructure crowns was made using the SEM method. This method is commonly used for a detailed analysis of the structure morphology of teeth [39], enamel and dentin in crowns [40] and hard tissues of the root [41]. However, some drawbacks need to be mentioned. Metwally et al. [42] pay attention to the sample preparation (coating) which can affect microscopy observations. In the studies, the samples were coated with a thin Au conductive layer. The SEM images presented in the previous section have shown greater heterogeneity of zirconia than porcelain. However, some surface irregularities were also found on the porcelain structure. The initial bacterial adhesion on the surface begins with surface irregularities (such as cracks, grooves). At these sites, bacteria are protected from natural removal forces and oral hygiene measures. What is more, the increase of roughness causes the increase of the surface available for bacterial adhesion. 

In order to assess the roughness of the materials used (zirconia and porcelain), profilometric tests were carried out for the samples not irradiated with diode laser and those after three variants of irradiation. Optical profilometry is a commonly used research method that enables the characterization of the material’s surface. This method enables the registration of three-dimensional images of the surface and determination of metrological parameters [43]. These parameters allow for a comprehensive assessment of the surface microgeometry of the tested materials. This technique is useful for testing such materials as porcelain and cubic zirconia used in dentistry. The surface texture, especially its roughness and waviness, can be characterized by many parameters, among which the most important are Ra, the arithmetic mean of the elevation profile, Rq, the mean square elevation profile, Rp, the height of the highest peak profile and Ry, the lowest recesses profile. There are many literature reports on the surface roughness of porcelain and zirconia used in dentistry [43,44]. The vast majority concern mechanical processing [45].

The research showed a different structure of the porcelain and zirconia surfaces. The highest roughness value Ra = 2.086 µm was obtained for the porcelain sample before the irradiation process for the largest scan 946 µm × 1261 µm. Such high roughness parameters can result from the accumulation of a large number of pits in this area. For the remaining scan sizes, 117 µm × 156 µm and 46.9 µm × 62.5 µm, the roughness parameters were much smaller, reaching a minimum value of Ra = 0.141 µm.

In the case of zirconia, a much larger number of pits were observed on the surface, but a much smaller depth, up to 8 µm. The research proving that porcelain is rougher compared to zirconia is consistent with the literature reports [44]. The highest roughness value of Ra = 1.100 µm was obtained for the zirconia sample after irradiation L4 for the largest scan 946 µm × 1261 µm. For the remaining scan sizes, the roughness parameters were much smaller, reaching the minimum value of Ra = 0.278 µm.

The most important conclusion from the research is the fact that the laser operation, regardless of the time, does not have a destructive effect on the tested material. No significant differences were observed in the roughness parameters before and after irradiation. This was confirmed by the optical microscopy tests, SEM tests and optical profilometry.

## 5. Conclusions

The use of a multidisciplinary approach to the implementation of this research allows us to state that radiation with a diode laser with a wavelength of 810 nm and a power of 3.84 W in pulsed mode is suitable for the decontamination of zirconium and porcelain crowns contacted directly with the perimplantation area. The tested variants of laser irradiation in each case statistically significantly reduced the number of microorganisms. The percentage reduction in the number of microorganisms is proportional to the number of repetitions of the irradiation. This method, in the tested laser power parameters and with the use of a specific exposure time, effectively reduces microorganisms and does not deteriorate the properties of the structure and morphology of the zirconium surface and porcelain used for the production of crowns as a superstructure of dental implants. This was confirmed by research using both microscopic techniques and optical profilometry. Subtle differences on the surface of the tested materials indicate their heterogeneity. 

Safe elimination of microorganisms, including pathogens, from places in the vicinity of dental implants through diode laser irradiation may support the treatment of peri-implant inflammations and other tissue infections, which may accelerate patient recovery.

## Figures and Tables

**Table 1:** The average concentration of microorganisms on the zirconia crowns, porcelain crowns and on the teeth surface with the interval of 6 months (detected by the culture dependent technique).

Type of Sample	Number of Microorganisms [CFU/0.4 cm^2^]		
	Zirconia Crown	Porcelain Crown	Tooth
I (initial test)	1.5 × 10^7^ ± 1.1 × 10^7^	1.7 × 10^5^ ± 1.8 × 10^4^	1.0 × 10^6^ ± 2.8 × 10^5^
II (after 6 months)	3.5 × 10^7^ ± 8.9 × 10^6^	1.4 × 10^7^ ± 5.4 × 10^6^	6.0 × 10^6^ ± 5.2 × 10^6^

Mean ± standard deviation.

**Table 2:** The average number of the most abundant species of microorganisms on the surfaces of individual materials identified by the MALDI-TOF MS.

Microorganism	Number of Microorganisms [CFU/0.4 cm^2^]		
	Zirconia Crown	Porcelain Crown	Tooth
*Rothia dentocariosa*	9.0 × 10^6^ ± 8.17 × 10^5^	3.4 × 10^6^ ± 1.77 × 10^6^	1.2 × 10^6^ ± 5.09 × 10^4^
*Rothia aeria*	2.0 × 10^6^ ± 5.09 × 10^4^	8.0 × 10^5^ ± 1.54 × 10^4^	2.0 × 10^5^ ± 3.42 × 10^4^
*Neisseria subflava*	5.6 × 10^6^ ± 2.52 × 10^5^	3.0 × 10^6^ ± 5.09 × 10^4^	0.0 × 10^0^ ± 0.00 × 10^0^
*Streptococcus pneumonie*	8.4 × 10^7^ ± 7.80 × 10^6^	3.2 × 10^7^ ± 2.53 × 10^6^	3.0 × 10^6^ ± 1.77 × 10^6^

Mean ± standard deviation.

**Table 3:** The identification results of microorganisms isolated from the surfaces of teeth zirconia crowns and porcelain crowns using the NGS method.

Group of Microorganisms and Relative Abundance	Identified Microorganisms Taxa		
	Zirconia Crown	Porcelain Crown	Teeth
Bacteria >2.00%	*Enterococcaceae* unid. *Rothia mucilaginosa* *Streptococcus* unid.	*Enterococcaceae* unid. *Rothia mucilaginosa* *Streptococcus* unid.	*Bacillus* unid. *Rothia mucilaginosa* *Staphylococcus aureus* *Streptococcus* unid.
Bacteria <2.00%	*Abiotrophia* unid. *Actinomyces* unid. *Atopobium* unid. *Campylobacter* unid. *Capnocytophaga* unid. *Corynebacterium durum* *Erwinia* unid. *Fusobacterium* unid. *Granulicatella* unid. *Gemellaceae* unid. *Haemophilus parainfluenzae* *Lautropia* unid. *Lactobacillales* unid. *Leptotrichia* unid. *Micrococcaceae* unid. *Neisseria subflava* *Porphyromona* *Prevotella melaninogenica* *Prevotella nigrescens* *Prevotella pallens* *Rothia aeria* *Rothia dentocariosa* *Rickettsiales* unid. *Staphylococcus aureus* *Streptococcus anginosus* *Streptophyta* unid. *Veillonella dispar*	*Actinomyces* unid. *Corynebacterium durum* *Enterobacteriaceae* unid. *Fusobacterium* unid. *Gemellaceae* unid. *Granulicatella* unid. *Haemophilus parainfluenzae* *Kocuria* unid. *Lactobacillales* unid. *Micrococcaceae* unid. *Neisseria subflava* *Prevotella nigrescens* *Rothia aeria* *Rothia dentocariosa* *Staphylococcus aureus* *Veillonella dispar*	*Actinomyces* unid. *Bacillaceae* unid. *Corynebacterium durum* *Enterobacteriaceae* unid. *Erwinia* unid. *Granulicatella* unid. *Haemophilus parainfluenzae* *Lautropia* unid. *Neisseria subflava* *Planococcaceae* unid. *Rothia aeria* *Rothia dentocariosa* *Veillonella dispar*
Fungi >2.00%	*Aureobasidium pullulans* *Cladosporium halotolerans* *Malassezia restricta* *Mariannaea pinicola* *Nectria ramulariae* *Penicillium thomii*	*Cladosporium delicatulum* *Malassezia restricta* *Mariannaea pinicola* *Nectria ramulariae* *Penicillium thomii* *Pezicula sporulosa* *Trametes gibbos*	*Cladosporium delicatulum* *Malassezia arunalokei* *Malassezia restricta* *Mariannaea pinicola* *Nectria amulariae* *Penicillium thomii*

Unid.—OTU identified to the level of order or family, unidentified to the species level.

**Table 4:** The results of the effectiveness of laser irradiation against the most common microorganisms on the porcelain crowns, zirconia crowns as well as teeth before and after irradiation with various laser dose.

Species of Microorganism	Material	Number of Microorganisms [CFU/0.4 cm^2^]	Reduction in the Number of Microorganisms after Irradiation [%]		
		Unirradiated	L1	L2	L3
*Streptococcus pneumonie*	Zirconia crown	2.0 × 10^9^ ± 2.8 × 10^8^	98.30 *	97.36 *	99.08 *
	Porcelain crown	6.9 × 10^8^ ± 5.9 × 10^7^	53.26 *	80.33 *	84.57 *
	Tooth	7.3 × 10^8^ ± 5.8 × 10^7^	99.70 *	98.73 *	99.91 *
*Rothia dentocariosa*	Zirconia crown	2.6 × 10^5^ ± 6.3 × 10^4^	56.09 *	61.99	62.45 *
	Porcelain crown	3.6 × 10^5^ ± 3.1 × 10^4^	76.26 *	87.66 *	89.23 *
	Tooth	1.6 × 10^6^ ± 9.1 × 10^4^	88.35 *	92.90 *	98.77 *
*Neisseria subflava*	Zirconia crown	1.0 × 10^7^ ± 1.4 × 10^6^	69.65 *	87.11 *	87.25 *
	Porcelain crown	9.9 × 10^6^ ± 5.3 × 10^5^	91.26 *	98.13 *	99.84 *
	Tooth	1.1 × 10^6^ ± 7.9 × 10^4^	44.24 *	44.60 *	51.08 *
*Staphylococcus aureus* ATCC	Zirconia crown	3.4 × 10^8^ ± 4.7 × 10^7^	81.38 *	97.60 *	99.87 *
	Porcelain crown	1.6 × 10^8^ ± 2.0 × 10^7^	99.92 *	99.74 *	99.94 *
	Tooth	9.6 × 10^7^ ± 3.4 × 10^6^	99.94 *	72.81 *	100.00 *

* statistically significant difference in reference to the control sample; ANOVA and LSD at a significance level *p* < 0.05.

**Table 5:** The roughness parameters for the porcelain samples before and after irradiation.

Scan Size	Ra for Samples [µm]			
	Unirradiated	After Irradiation		
		L1	L2	L3
946 µm × 1261 µm	2.086 ± 0.20	1.182 ± 0.11	0.926 ±0.08	1.166 ± 0.11
117 µm × 156 µm	0.452 ± 0.04	0.330 ± 0.03	0.293 ± 0.02	0.406 ± 0.03
46.9 µm × 62.5 µm	0.204 ± 0.01	0.141 ± 0.03	0.192 ± 0.01	0.143 ± 0.01

**Table 6:** The roughness parameters for the zirconia samples before and after irradiation.

Scan Size	Ra for Samples [µm]			
	Unirradiated	After Irradiation		
		L1	L2	L3
946 µm × 1261 µm	0.795 ± 0.07	0.939 ± 0.09	0.477 ± 0.04	1.100 ± 0.10
117 µm × 156 µm	0.337 ± 0.03	0.394 ± 0.03	0.377 ± 0.03	0.440 ± 0.04
46.9 µm × 62.5 µm	0.310 ± 0.02	0.278 ± 2.02	0.281 ± 0.02	0.350 ± 0.03
